# Potential factors affecting the impact of community reinforcement and family training. Secondary analysis of an RCT

**DOI:** 10.1186/s12889-024-17656-1

**Published:** 2024-01-17

**Authors:** Rikke Hellum, Randi Bilberg, Anna Mejldal, Anette Søgaard Nielsen

**Affiliations:** 1 The Unit of Clinical Alcohol Research (UCAR), Institute of Clinical Research, University of Southern Denmark, J.B. Winsløws vej 18, Odense C, 5000 Denmark; 2 Department of Mental Health Odense, Region of Southern Denmark, J.B. Winsløws vej 18, Odense C, 5000 Denmark; 3 Odense Patient Data Explorative Network (OPEN), Odense University Hospital, J.B. Winsløws vej 9A, Odense C, 5000 Denmark; 4 Department for Data, Innovation and Research, Lillebaelt Hospital, University Hospital of Southern Denmark, Vejle, Denmark

**Keywords:** CRAFT, Community reinforcement and family training, Concerned significant others, Relatives, Addiction, Addiction treatment, Alcohol problems, Alcohol treatment

## Abstract

**Background:**

In addition to increasing the quality of life among concerned significant others (CSOs), Community Reinforcement and Family training (CRAFT) aim at helping CSOs motivate treatment-refusing identified patients (IPs) into treatment through a positive reinforcement process. The aim of the present study was to investigate if the following factors, measured at baseline, have an influence on IP future treatment engagement (1) Type of relation between CSO and the IP (2) The amount of time the CSO spend with the IP (3) if the IP knows that the CSOs seeks help, and (4) The CSO’s own alcohol use.

**Methods:**

A secondary analysis from the Danish CRAFT study. CSOs completed a self-administered questionnaire at baseline, after three months, and six months. To investigate the relationship between the four variables and treatment engagement, logistic regression was used.

**Results:**

CSO’s relation to the IP, the frequency of contact between the CSO and the IP, and the CSO’s AUDIT score at the time of the baseline interview were not associated with the IP’s treatment engagement. If CSO at baseline had informed the IP that the CSO participated in CRAFT, odds for IP treatment engagement were significantly higher (adjusted OR [(CI)] = 2.29 [1.13; 4.63] (*p* < 0.05), relative to if IP not being informed.

**Conclusions:**

CRAFT has a higher impact on the likelihood for treatment seeking, if the CSOs inform the IP about his or her own help seeking in order to change the situation. The underlying mechanism behind this is needs further investigations.

## Background

As in many other Western countries, alcohol use is common in Denmark. More than 85% of adults in Denmark consume alcohol [1]; it is estimated that 585,000 Danes have a harmful use and 148,000 Danes (equaling 3% of the population) is dependent of alcohol [2]. Alcohol use disorders (AUD) can have serious consequences for both the drinkers (or so-called identified patients, IPs) [3, 4] and their concerned significant other (CSO). The impact on the CSO varies, depending on type of relation to the IP; partners who live together with the IP experience other problems than do e.g., siblings, adult children, and friends [5].

Partners to IPs live in a daily stressful situation and are often exposed to aggressions, psychological and sometimes physical abuse from the IPs which leads to frequent conflicts [6]. Thus, partners to IPs report lower quality of life than the general population [7], even if they do not live together [7]. Moreover, relationsship distress and AUD are strongly related and it has been shown that one partner’s AUD influence the other partners use of alcohol in a negatively way [8]. Futhermore, parents of IPs often feel inadequate in their partenting [9] which can lead to physiological and/or physiological symptoms [10]. Children of IPs frequently experience verbal abuse, neglect, being left alone unsupervised, and having to adopt responsibilities or parenting roles at an early age [11, 12]. This can affect the child during all stages of development and make them at risk for developing, for example, behavioral problems, emotional difficulties, behavioral disturbance and social isolation and these problems might follow them as adolescents and into adulthood [12]. Unlike family, friends can be chosen and abandoned, making these relationships more dynamic. The interaction between friends is often less regular, less continuous and usually less intensive, seen over a lifespan, which make the commitment towards friends less demanding in relation to time, emotions, finance and responsibilities [13].

Despite of the negative effects alcohol causes, CSOs are highly concerned and worried for the IP’s health and wellbeing [10, 14]. Therefore, CSOs are often highly motivated to help the IP to become sober or to a reduction in drinking [15]. The CSOs hold important knowledge about the IP since some of the CSO spend considerable time with the IP, and this gives the CSOs a possibility to influence the IP in a higher degree than a treatment provider who might see the IP one hour per week [16]. The CSO can also play an important role in recovery of the person with AUD by supporting and participating in the alcohol treatment. There is evidence which shows that when the CSOs participate in the IP’s treatment, the treatment outcome is better [8].

Often a CSO has tried to help the IP in several ways and during a long time, but it can be a struggle if the IP has not acknowledged the alcohol problem or motivated for treatment [15]. The program Community Reinforcement and Family training (CRAFT) aims at helping CSOs of treatment-refusing IPs into treatment through a positive reinforcement process but also to increase the CSOs’ quality of life and the relation between to the IP [15]. So far, a CRAFT intervention offered to the CSO is the method that has been most evident in increasing the likelihood that the IP will enter treatment [17].

Earlier studies on CRAFT indicate that the most common CSOs seeking the program were partners/spouses, followed by adult children. In some studies, also siblings and parents participated to a minor degree [17]. Only one study on CRAFT has identified an association between ‘type of relation’ between the CSO and the IP, and IP treatment entry; Meyers, Miller [15] found that parents were more able to engage the IP adult child in treatment than non-parents [15, 17]. The IPs in this study were, however, drug users.

Most studies on CRAFT have had rather rigorous inclusion criteria according to how much contact the CSO and IP should have. For example, in some studies it was an inclusion criteria that the CSO and IP should spend 40% of their time together [18–20], or they should be spending at least 20 h together per week or being living together [21], or see each other a least 12 days per month [22]. Furthermore, several studies on CRAFT have excluded CSOs who themselves had indications on alcohol use disorder or other substance use disorder [18, 21–24].

Hence, there is limited knowledge about what characterize the CSOs who succeed in motivating their IPs to treatment. In a recent Danish study on CRAFT, to execute the study as close as possible to ‘real-world’-practice and thus increase the ecological validity, the inclusion criteria were limited to as few as possible; there were, for example, no requirement to amount of time that the CSOs spend with the IPs. Instead information on ‘time spend together’ were collected [25]. Neither were there any requirements to the type of relation between the CSO and the IP (i.e. partner, friend, parent, child). Thus, the Danish study allows for investigating if there is a correlation between time spend with the IP and IP treatment entry, if type of relation between the IP and the CSO is of importance and if the CSO’s own drinking had an impact, which is the aim of the present study.

By means of descriptive explorative analysis we wish to investigate if the factors normally restricted by means of inclusion and exclusion criteria have an influence on IP treatment engagement three or six months after the CSOs enrolled in the CRAFT study: (1) Type of relation between CSO and the IP at the time when the CRAFT intervention is initiated (baseline) (2) The amount of time the CSO spend with the IP at the time when the CRAFT intervention is initiated (baseline) (3) The CSO’s own alcohol use, measured by means of Alcohol Use Disorder Identification test (AUDIT) at the time when the CRAFT intervention is initiated (baseline). Furthermore, we wished to explore if it was of importance that the IP knew that the CSOs entered the CRAFT intervention, since the therapists in the Danish CRAFT-study got the impression that informing the IP added an additional albeit gently pressure on the IP and impacted on the outcome [26].

## Materials and methods

The present study is a secondary analysis of data from the Danish study of the CRAFT conducted from 2017 to 2019 [25]. The aim of the original study was to compare CRAFT in three formats: CRAFT delivered by the means of 6 individual sessions, or by the means of CRAFT delivered as 6 sessions of CRAFT as OPEN group format, or CRAFT delivered as a self-help book only (control group) [27]. The study, which included 255 CSOs did not show any statistic significant difference between the three interventions group according to treatment entry for the IP or change in the CSOs’ quality of life and depression score [25].

In the original study, 18 treatment centers were randomized to deliver CRAFT in one of the three formats. (1) s six individual sessions supported by written material. (2) As six open group sessions, supported by written material. (3) Self-administered format and by means of written material only [25]. CSOs were eligible to participate in the study if they approached the treatment facility expressing concern for their IP’s drinking habits and were not already in treatment or had received treatment in the past three months. The CSOs were not told beforehand which intervention each facility had been allocated to [25]. For more information about the randomization and intervention please see the primary outcome article [25].

### Participants

#### Recruitment

The recruitment of the CSOs and the interventions were conducted between January 1st, 2018, and December 31st, 2019. The intervention was targeting all CSO and not only partners or parents, who fitted the inclusion and exclusion criteria below. To disseminate the information on CRAFT interventions being available to the public and the possibility of CSOs needing to seek it, the participating local authorities distributed information leaflets and posters were and used advertisements in local newspapers and videos and posts on social media, linked to the alcohol treatment centers’ websites and their Facebook pages. Further, information about the project was posted on national websites for counseling on alcohol problems such as Alkohol & Samfund (in English: “Alcohol & Society”) and the National Telephone Hotline ‘Alkolinjen’ [28].

#### Inclusion criteria (CSO)

Any individual with a close relationship to someone, which they considered suffered from AUD, could participate in the trial if they met the following criteria: (1) 18 years or older; (2) being a CSO with concern for an IP’s drinking habits; [3] not currently receiving treatment for an alcohol problem; (4) have the intention of maintaining contact with the center for the next 90 days; (5) have had regular contact with the IP for the past 90 days (face-to-face contact for several hours on, at least, a weekly basis) or the desire to re-establish regular contact with an IP; and (6) being prepared, at least to some extent, to support the IP if they should choose to seek treatment [25].

#### Exclusion criteria (CSO)

CSOs were excluded only if they (1) suffered from dementia or other cognitive disorders; (2) did not speak Danish; (3) were psychotic or otherwise severely mentally ill; (4) had been receiving treatment for alcohol problems for the past three months; and (5) were concerned about a person who, according to the CSO, mainly used illegal substances [25].

### Assessments

After enrollment and before the first session, the CSOs completed a self-administered questionnaire (baseline, t0) on a tablet, starting with an informed consent form. Data were collected again after three months (t1) and six months (t2) by a self-administered web-based battery of questionnaires, emailed to them by secure email, or by telephone interview, depending on their preferred choice. The participants received up to three reminders for the follow-up questionnaire [25]. The study aimed at supporting the CSOs and no information was collected from the IP, since the research team was not in contact with the IP. Thus, information about the IP was based on information from the CSO.

### Measures and variables

#### Demographics

Demographic information included gender and age.

#### Additional information on the CSO

At baseline, we asked the CSOs to state the kind of relation they had to the IP. This variable was recoded into four categories, partner, daughter/son, parent, and others (covering neighbours, friend, other, do not know). Furthermore, we asked how much contact they have with the IP. This variable was recoded into three categories: Daily/almost daily contact covering the CSOs who answered daily or 4–5 times a week. Weekly contact covering the CSOs who answered 2–3 times a week or 1–2 times a week and monthly or no contact covering the CSOs who answered 1–2 times a month or no contact the last four weeks. We also asked if they had told their IP that they had entered the CRAFT intervention (yes/no). Finally, the Alcohol Use Disorder Identification Test (AUDIT) [29] was used to collect information on the CSOs’ use of alcohol. Response options for each question were scored from 0 to 4 and added up. The total score was classified into two groups: <8 (no risky alcohol use) or > = 8 (risky alcohol use).

The intervention, that the CSOs received, was labeled (1) Individual session (2) Group sessions (3) Self-administered.

#### Outcome measures

The outcome was measured during the follow-up interview with the CSOs at three and six months after entering the study, based on the question: “Has the IP entered treatment for his/her alcohol use?”. The CSO could answer either yes, no or do not know. The information was defined as missing if the CSOs had participated in no follow-up interviews and thus not answered the question at any time. The information was dichotomized into 1 if the CSO answered positive (yes) at least one follow-up, versus no positive answers at any follow-up.

### Statistical analysis

We used descriptive statistics to report demographic data, relationship type, contact frequency, IP’s knowledge of CSO’s participation and the CSO’s AUDIT score, using mean (SD) for continuous variables and numbers (percent) for categorical variables. Difference by dropout status were compared between groups using Pearson’s Chi2 tests for categorical variables and t-tests for continuous variables.

All analysis was based on complete data. To investigate the relationship between the binary outcome and each of the four explanatory variables, logistic regression was used. In the first analysis, we only adjusted for the type of intervention the CSO received. In the second analysis, we additionally adjusted for gender and age of the CSO. Results are reported as odds ratios (OR) and 95% confidence intervals.

Finally, we performed a sensitivity analysis by coding all missing outcomes as negative, indicating that the IP did not enter treatment.

### Ethics approval and consent to participate

This study was approved by the Danish Data Protection Agency (Region of Southern Denmark 2008-58-0035 project no. 17/46074). The study was submitted for ethical approval to the Danish Ethics Committee (Project-ID: S-20170148) but according to Danish law, the study did not require formal approval since it was a questionnaire survey to compare different ways of implementing a recommended treatment method, CRAFT, according to the National Clinical Guidelines in Denmark.

All participants were informed, both orally and in writing, about the procedures for attending the study. The participants signed an informed consent document prior to participating in the study. All relevant guidelines have been followed according to the Declaration of Helsinki.

The study was registered at ClinicalTrials.gov Identifier: NCT03281057.

## Results

### Study sample

During the study period from 2018 to 2019, a total of 255 CSOs were included in the study. Among the 249 participants in the study, 60% (*n* = 151) completed the three months follow-up assessment. At six months follow-up, 55% (*n* = 136) of all the participants completed the questionnaire.

#### Sample

Baseline characteristics of the CSOs and IPs and a drop-out analysis are presented in Table 1. The CSOs were mostly female (85% *n* = 214), and the mean age of the participants was 49.0 (SD; 13.9). The most common relation to the IP was partner/spouse (49%, *n* = 124), the second most common was parent (22%, *n* = 56), and the third most common was adult child (12%, *n* = 30). The majority of the CSOs had daily contact with the IP (58%, *n* = 149), and the rest of the CSOs had weekly contact (20%, *n* = 52) or monthly contact (21%, *n* = 54). More than half of the CSOs (57%; *n* = 146) had told the IP that they participated in CRAFT. The CSOs’ AUDIT mean score was 4.6 (SD: 3.4) and 16% (*n* = 40) had an AUDIT score of or above eight, and thus screening positive for risky alcohol use. Only three CSOs scored 15 or above on the AUDIT. There were a few missing responses of AUDIT (*n* = 10). The dropout analysis was made for those who did not complete the three- or six- months follow-up. The analysis showed that the ones who did not answer at three- or six-months follow-up were younger.

**Table 1:** Baseline characteristics of CSOs at baseline, by completion of study participation or drop-out

Factor	Level	Total sample	Responders*	Non-responders**	p-value***
N		255	170	85	
Gender	Men	39 (15%)	25 (15%)	14 (17%)	0.65
	Women	214 (85%)	145 (85%)	69 (83%)	
CSO’s age: mean (SD)		49.0 (13.9)	50.7 (13.2)	45.6 (14.9)	0.006
Randomization	Self help	68 (27%)	42 (25%)	26 (31%)	0.22
	Group	90 (35%)	57 (34%)	33 (39%)	
	Individual	97 (38%)	71 (42%)	26 (31%)	
Contact frequency	Daily	149 (58%)	100 (59%)	49 (58%)	0.84
	Weekly	52 (20%)	33 (19%)	19 (22%)	
	Monthly/never	54 (21%)	37 (22%)	17 (20%)	
Relation, the IP is my:	Partner	124 (49%)	88 (52%)	36 (42%)	0.26
	Child	30 (12%)	16 (9%)	14 (17%)	
	Parent	56 (22%)	35 (21%)	21 (25%)	
	Other	45 (18%)	31 (18%)	14 (17%)	
The IP know that the CSO participate in CRAFT	Does not know/unsure	146 (57%)	93 (55%)	53 (62%)	0.24
	Knows	109 (43%)	77 (45%)	32 (38%)	
AUDIT score, mean (SD)		4.6 (3.4)	4.7 (3.5)	4.5 (3.2)	0.74
AUDIT score	< 8	205 (84%)	140 (85%)	65 (81%)	0.47
	≥ 8	40 (16%)	25 (15%)	15 (19%)	

* Responders is defined as having participated in at least one follow-up

** Non-responders are defined as having participated in the baseline interview and started the intervention, but not participated in any follow-up interviews

*** Testing difference between responders and non-responders

### Factors influencing on IP treatment engagement

**Fig. 1:**
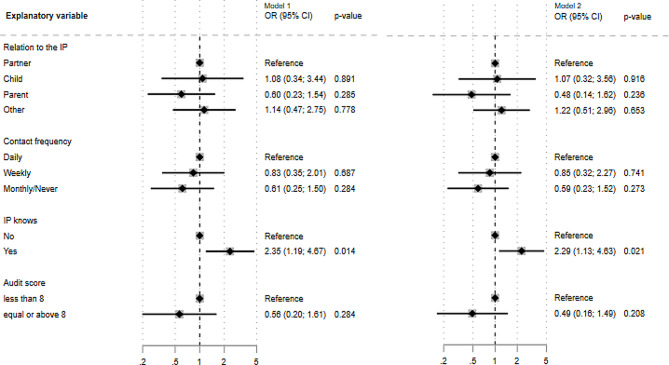
Logistic regression results of relation to the IP, contact frequency, IP knows that CSO participate in CRAFT, and CSO’s AUDIT score and influence treatment Engagement*. *Model 1 adjusting for type of intervention. Model 2 adjusting for age and gender

The results from the logistic regression models are shown in Fig. 1. It can be seen from the adjusted model (model 2) that the CSO’s relation to the IP was not associated with the IP’s treatment engagement. Relative to partners (reference group) the odds ratio for adult children was (OR) [95% confidence interval (CI)] = 1.07 [0.32; 3.56], for parent: OR [(CI)] = 0.48 [0.14; 1.62], and for other kinds of relation OR [(CI)] = 1.22 [0.51; 2.96]. The frequency of contact between the CSO and the IP was not significantly associated with treatment engagement, although the tendency was that a lower contact frequency meant lower probability of treatment engagement. Compared ‘daily contact’ (reference group), the CSOs who had only weekly contact had OR [(CI)] = 0.85 [0.32; 2.27], and the OR for those who reported monthly/never contact was 0.59 [0.23; 1.52]. The CSO’s AUDIT score was also not significantly associated with treatment engagement. Compared to having an AUDIT score below 8, having an AUDIT score equal or above 8 led resulted in a 50% lower odds of treatment engagement (OR [(CI)] = 0.49 [0.16; 1.49]), however this was also not statistically significant. In both the adjusted and un-adjusted models, it can, however, be seen that the odds for IP treatment engagement was significantly higher. If the IP knew that the CSO participated in CRAFT the odds of the IP entering treatment doubled (adjusted OR [(CI)] = 2.29 [1.13; 4.63] (*p* < 0.05) compared to the situation where the IP did not know.

The sensitivity analysis where all missing outcomes were coded as negative (results not shown), indicating that the IP did not enter treatment did not affect the results. The results maintained the same trends, and significance persisted only when the intervention participant was aware of the CSO’s involvement in the CRAFT program.

## Discussion

The present sub-study investigated if (1) type of relation between the CSO and the IP (2) the amount of time the CSO spend with the IP (3) the CSO’s own alcohol use, measured by means of AUDIT, and (4) IP knowing that the CSOs has sought the CRAFT intervention, had an influence on whether the IP engaged in treatment within three or six months after the CSOs enrolled in the CRAFT study. We found that if the CSO had informed the IP that they sought professional help, the CRAFT the odds were 2.25 time higher that the IP had entered treatment three or six months after the CSO enrolled in the CRAFT study.

A study of Meyers and colleagues found that parents were particularly effective in motivating their adolescences or adult children in seeking treatment after having themselves participated in a CRAFT-intervention [15]. However, similar to other studies [17], we did not find any association between type of relation to the IP and treatment engagement rate, neither did we find an association between contact frequency and IP treatment engagement. Most previous CRAFT studies were designed with strict inclusion criteria regarding time spend together [18–20]. The rationale for the criteria on time spend together was that the CSOs is more able to impact the IP if they spend more time together [16]. However, the results from the present study does not support that the CSOs has more success in engaging the IP if they spend more time with the IP. From a clinical perspective, it can thus be argued that CSOs should be offered participation in a CRAFT program if the CSO him or herself judges that it might be meaningful in view of the CSO’s relation and time spend with the IP, rather than strict criteria for enrollment.

In the present study, we saw a tendency towards CSOs with an AUDIT score of eight or more and thus screening positive for at-risk drinking, having less chance of motivating their IP to treatment. However, this tendency was small and not significant. Earlier studies on CRAFT excluded participants if they met the criteria for an alcohol or other substance use disorder [18, 21, 23, 24, 30], but the present findings indicates that it might be relevant to include CSOs with at risk-drinking in future studies of interventions of CRAFT. However, more research is needed in order to draw firm conclusions.

The most striking finding in the present study was that if CSO had informed the IP that they participated in CRAFT, the odds for the IP entering treatment within 3 or 6 months were 2.25 time higher, compared to the situations where the CSOs sought help in secrecy. There may be several explanations on this finding. During the CRAFT intervention, it was entirely up to the CSOs to decide if they wished to inform the IP or not about their engagement in CRAFT. However, some therapist recommended informing the IP, if the CSO was ready for it [26]. From an ethical perspective, it may seem appropriate always to inform the IP, but from a safety perspective there may be good reasons for the CSOs not to inform. Nevertheless, informing the IP might indicate that CSOs who are able or in a position to have a more open dialogue with the IP, also are able to change the balance of the relationship in a direction that impacts IPs’ motivation to treatment seeking, i.e., demonstrating that the CSOs both care about the IP and takes responsibility for own situation. It may signal to the IP that the CSO is serious in his or her worry or dissatisfaction with the situation. It can also be hypothesized that the CSO informing the IP about the decision to help-seeking may be a sign on a relationship that is already based on mutual respect and trustworthy communication, and that it is such overall quality of a relationship that impacts on the IPs’ decisions about treatment seeking. Another explanation might be, that CSOs not telling their IP that they participate in a CRAFT intervention, could be because they are relatively closer to breaking the relation to the IP. Seeking help may thus be a way to resolve ambivalence about whether to stay or leave, and a decision that the CSO feels he or she needs to take alone, but this is only guesswork. In contrast to this hypothetical explanation, it may also be that CSOs who have been open about help-seeking may feel more committed to engage in CRAFT and in motivating the IP. It is well known that commitment is strengthened if outspoken, behavior change is more likely [31].

In a prior, qualitative study of the experiences of the therapists, who performed the intervention in the Danish CRAFT study, the therapist considered that CRAFT worked better, if the CSO had told the IP that they participated in CRAFT [26]. Based on what the CSOs reported on homework assignments etc., the therapists found that when the CSOs had informed the IP, it allowed the CSO and IP to have a more open discussion about the CRAFT program and other issues related to the alcohol problem [26].

It was the aim of the Danish study of CRAFT to compare how three formats of delivering CRAFT interventions functioned in real life settings, i.e., operating Danish treatment institutions. Therefore, we had wide inclusion criteria to reflect the everyday situation. Our findings suggest that future studies should explore in more details what impact, type of relation, time spend with the IP and the alcohol use of the CSO has on outcome of CRAFT. The more knowledge, we have, the better foundation for future implementation of interventions like the present.

### Strengths and limitations

The present study is one of the largest studies on CRAFT. However, the study nevertheless did not include the number of CSOs that was expected and the findings in the present study should therefore be considered with caution. The study may be underpowered to detect associations in particular between time spend with the IP and IP treatment engagement, and between alcohol use of the CSO and IP treatment engagement. Furthermore, it may be regarded a limitation that the study is performed as a study of complete cases rather than on intention to treat. The study is characterized by a rather large number of non-responders on the follow-ups (dropouts) and we do not have information about outcome of this group. Although there were only few significant differences between responders and non-responders, the number of non-responders may have impacted on our findings. We performed a sensitivity analysis by coding all missing outcomes as negative (results not shown), indicating that the IP did not enter treatment. Despite this adjustment, the overall conclusion remained unchanged.

Strengths of this study were that we did not have narrow inclusion criteria according to time spend with the IP and type of relation. Instead, information about time spend with the IP was collected and thereby allowed for analysis. It is also a strength of the study that it is performed in operating alcohol treatment institutions and includes CSOs who have approached the institutions for help; the CSOs in the present study thus reflects the profiles of help-seeking CSOs that staff can expect to meet.

## Data Availability

The datasets generated and/or analysed during the current study are not publicly available due confidentiality and ethical reasons but are available from the corresponding author on reasonable request.

## Abbreviations

AUD: Alcohol use disorders
AUDIT: Alcohol Use Disorder Identification Test
CRAFT: Community Reinforcement and Family Training
CSO: Concerned significant other
IP: Identified Patient

## References

[1] Danish Health Authority. Danskernes Sundhed, Den Nationale Sundhedsprofil 2021. https://www.sst.dk/-/media/Udgivelser/2022/Sundhedsprofil/Sundhedsprofilen.ashx; 2021 10. marts 2022.

[2] Hansen AB, Hvidtfeldt UA, Gronbaek M, Becker U, Nielsen AS, Tolstrup JS (2011). The number of persons with alcohol problems in the Danish population. Scand J Public Health.

[3] Witkiewitz K, Litten RZ, Leggio L (2019). Advances in the science and treatment of alcohol use disorder. Sci Adv.

[4] Carvalho AF, Heilig M, Perez A, Probst C, Rehm J (2019). Alcohol use disorders. Lancet.

[5] Cameron D. Mapping the Social Consequences of Alcohol Consumption: Edited by Harald Klingemanna and Gerhard Gmel. WHO Regional Office for Europe/Kluwer Academic, Dordrecht. 2001, 170pp. hardback, £44.06. ISBN: 0-79236-740-5. Alcohol and Alcoholism. 2002;37(1):103-4.

[6] Orford J, Velleman R, Copello A, Templeton L, Ibanga A (2010). The experiences of affected family members: a summary of two decades of qualitative research. Drugs: Educ Prev Policy.

[7] Birkeland B, Foster K, Selbekk AS, Hoie MM, Ruud T, Weimand B (2018). The quality of life when a partner has substance use problems: a scoping review. Health Qual Life Outcomes.

[8] McCrady BS, Flanagan JC (2021). The role of the family in Alcohol Use Disorder Recovery for adults. Alcohol Res.

[9] Orford J, Velleman R, Natera G, Templeton L, Copello A (2013). Addiction in the family is a major but neglected contributor to the global burden of adult ill-health. Soc Sci Med.

[10] Orford J, Velleman R, Natera G, Templeton L, Copello A. Addiction in the family is a major but neglected contributor to the global burden of adult ill-health. Social science & medicine (1982). 2013;78:70– 7. 10.1016/j.socscimed.2012.11.036 23268776

[11] Laslett AM, Rankin G, Waleewong O, Callinan S, Hoang HT, Florenzano R (2017). A multi-country study of Harms to Children because of others’ drinking. J Stud Alcohol Drug.

[12] Velleman T (2007). Understanding and modifying the impact of parents’ substance misuse on children. Adv Psychiatr Treat.

[13] Pernanen K, Klingemann H, Gmel G (2001). Consequences of drinking to friends and the close social environment. Mapping the Social Consequences of Alcohol Consumption.

[14] Hellum R, Bilberg R, Nielsen AS. He is lovely and awful: the challenges of being close to an individual with alcohol problems. Nordic Stud Alcohol Drugs. 2021:145507252110448. 10.1177/14550725211044861 PMC8899274 35308468

[15] Meyers RJ, Miller WR, Hill DE, Tonigan JS (1998). Community reinforcement and family training (CRAFT): engaging unmotivated drug users in treatment. J Subst Abuse.

[16] Smith JEMR (2004). Motivating substance abusers to enter treatment: working with family members.

[17] Archer M, Harwood H, Stevelink S, Rafferty L, Greenberg N (2020). Community reinforcement and family training and rates of treatment entry: a systematic review. Addiction.

[18] Manuel JK, Austin JL, Miller WR, McCrady BS, Tonigan JS, Meyers RJ (2012). Community reinforcement and family training: a pilot comparison of group and self-directed delivery. J Subst Abuse Treat.

[19] Meyers RJ, Miller WR, Smith JE, Tonigan JS (2002). A randomized trial of two methods for engaging treatment-refusing drug users through concerned significant others. J Consult Clin Psychol.

[20] EÉk N, Romberg K, Siljeholm O, Johansson M, Andreasson S, Lundgren T (2020). Efficacy of an Internet-Based Community Reinforcement and Family Training Program to increase Treatment Engagement for AUD and to Improve Psychiatric Health for CSOs: a Randomized Controlled Trial. Alcohol & Alcoholism.

[21] Bischof G, Iwen J, Freyer-Adam J, Rumpf HJ (2016). Efficacy of the Community Reinforcement and Family Training for concerned significant others of treatment-refusing individuals with alcohol dependence: a randomized controlled trial. Drug Alcohol Depend.

[22] Kirby KC, Benishek LA, Kerwin ME, Dugosh KL, Carpenedo CM, Bresani E (2017). Analyzing components of community reinforcement and family training (CRAFT): is treatment entry training sufficient?. Psychol Addict Behaviors: J Soc Psychologists Addict Behav.

[23] Sisson RW, Azrin NH (1986). Family-member involvement to initiate and promote treatment of problem drinkers. J Behav Ther Exp Psychiatry.

[24] Miller WR, Meyers RJ, Tonigan JS (1999). Engaging the unmotivated in treatment for alcohol problems: a comparison of three strategies for intervention through family members. J Consult Clin Psychol.

[25] Hellum R, Bilberg R, Andersen K, Bischof G, Hesse M, Nielsen A (2022). Primary outcome from a cluster-randomized trial of three formats for delivering community reinforcement and family training (CRAFT) to the significant others of problem drinkers. BMC Public Health.

[26] Hellum R, Bilberg R, Nielsen AS. A qualitative study of the therapists’ experiences of working with Community Reinforcement and Family training. Submitted to Counselling and Psychotherapy Research: Linking research with practice. 2022.

[27] Hellum R, Nielsen AS, Bischof G, Andersen K, Hesse M, Ekstrom CT (2019). Community reinforcement and family training (CRAFT) - design of a cluster randomized controlled trial comparing individual, group and self-help interventions. BMC Public Health.

[28] Alkohol, Samfund. https://alkohologsamfund.dk/.

[29] Babor TF, Higgins-Biddle JC, Saunders JB, Monteiro MG (2001). Dependence WHODoMHaS. AUDIT: the Alcohol Use disorders Identification Test: guidelines for use in primary health care.

[30] Kirby KC, Marlowe DB, Festinger DS, Garvey KA, La MV (1999). Community reinforcement training for family and significant others of drug abusers: a unilateral intervention to increase treatment entry of drug users. Drug Alcohol Depend.

[31] Amrhein PC, Miller WR, Yahne CE, Palmer M, Fulcher L (2003). Client commitment language during motivational interviewing predicts drug use outcomes. J Consult Clin Psychol.

